# Perceptions of eHealth-Enabled Physical Activity Interventions Among Cancer Survivors: Mixed Methods Study

**DOI:** 10.2196/16469

**Published:** 2020-04-28

**Authors:** Ciaran Haberlin, Dearbhaile M O' Donnell, Jonathan Moran, Julie Broderick

**Affiliations:** 1 Discipline of Physiotherapy School of Medicine, Trinity College Dublin University of Dublin Dublin Ireland; 2 Department of Medical Oncology St. James's Hospital Dublin Ireland

**Keywords:** cancer, eHealth, exercise, focus group, physical activity, qualitative, mobile phone

## Abstract

**Background:**

Achieving adequate levels of physical activity (PA) is especially important for cancer survivors to mitigate the side effects of cancer and its treatment as well as for other health benefits. Electronic health (eHealth)-based PA interventions may offer feasible alternatives to traditionally delivered programs and optimize physical recovery after a cancer diagnosis, but perspectives of cancer survivors on this new delivery medium have not been extensively explored.

**Objective:**

The overall aim was to explore participants’ perspectives of eHealth-enabled PA interventions to inform the design of a future intervention among cancer survivors.

**Methods:**

The study took place in a designated cancer center in Dublin, Ireland. A preceding questionnaire-based study was conducted primarily to establish interest in participating in subsequent eHealth-based studies. A follow-on focus group study was conducted to explore the concept of eHealth-based PA interventions for cancer survivors. The data were analyzed using thematic analysis.

**Results:**

The questionnaire-based study (N=102) indicated that participants had a high level of interest in participating in follow-on eHealth-based studies. The focus group study (n=23) indicated that, despite some trepidation, overall positivity was expressed by participants toward the concept of eHealth-based PA interventions. Four themes were generated: (1) Health impact, including PA as a barrier and as a motivating factor, (2) Education needs, which emphasized the need for integrated information about PA and to increase technical literacy, (3) Goal setting, which should be integrated within the technical specification as a motivating factor, and (4) Support needs, as well as the importance of personalized human interaction, in tandem with technology.

**Conclusions:**

Qualitative research at the pretrial phase adds value to the design of a complex intervention and is especially useful in an area such as eHealth. The findings highlighted an interest in participating in eHealth-focused research as well as barriers, training needs, and key design features that can be applied to optimize the design of future eHealth-based PA interventions in cancer.

## Introduction

The benefits of physical activity (PA) in cancer patients are well known, including improvements in quality of life, improvement in function, and a possible reduction in risk of recurrence in some cancer types [1]. Despite those known benefits, uptake of PA by cancer survivors is low from the time of diagnosis through to survivorship [2,3]. The challenge remains to elucidate the optimal type of intervention for increasing PA levels in cancer survivors. The majority of PA interventions in cancer survivors are low-tech and delivered face-to-face in a group setting which is time- and resource-intensive, and accessibility can be limited [4,5]. Alternative models of delivery are warranted. The emergence of increasingly sophisticated technologies, with the potential to enhance the delivery of PA interventions, may provide a feasible and scalable alternative to traditional interventions [6].

Usage of electronic technologies in the general population is high. The number of smartphone users worldwide currently exceeds three billion and is predicted to increase further over the coming years. China, India, and the United States have the highest number of smartphone users with each country exceeding the 100 million user mark [7]. In the United Kingdom, 45.1 million people used the internet on a daily basis in 2019 according to the UK Office for National Statistics, beating the record set in 2016 [8]. Despite the ubiquity of smartphones and their high usage, harnessing their benefits for health benefits is relatively new.

There is emergent systematic review-level evidence in favor of the health benefits of electronic health (eHealth) interventions. eHealth is a concept in health care that may present opportunities to improve PA in cancer survivors. eHealth has been defined as “health services and information delivered or enhanced through the Internet and related technologies” and eHealth “characterizes not only a technical development, but also a state-of-mind, a way of thinking, an attitude, and a commitment for networked, global thinking, to improve health care locally, regionally, and worldwide by using information and communication technology” [9].

A systematic review including almost 5000 participants indicated the promise of using mobile apps and SMS text messaging as mobile health (mHealth) interventions, with studies showing an improvement in physical health and significant reductions of anxiety, stress, and depression [10]. Similarly, a further systematic review indicated the potential of apps in improving symptom management through self-management interventions in long-term conditions [11], although little is known about their economic benefit and long-term sustainability.

Only a small number of studies have integrated eHealth as a delivery medium for PA interventions in cancer survivors [12]. New types of health service interventions can be complex [13] and difficult to integrate into practice. The Medical Research Council (MRC) has proposed a framework for the development of complex interventions [14]. This phased approach of health service evaluation begins with a theoretical element, then integrates a series of preliminary studies to inform the design of an intervention element. Integrating qualitative research can optimize the robustness of interventions [15], and this approach has been utilized within a number of complex interventions in the pretrial design phase [13,16,17].

Aligned to the recommendations of the MRC framework [14], we first conducted a systematic review of eHealth-based PA interventions [12]. This review identified only 10 studies, which included eHealth-based PA programs across a diversity of platforms. We found that consensus is lacking in terms of the optimal eHealth-based intervention design in the cancer setting.

Although previous studies have explored perspectives of cancer survivors toward exercise, these studies have mainly been conducted after completion of a structured exercise program [18-21]. One of the disadvantages of gaining participant perspectives after completion of an intervention are that preferences are influenced by their direct experience of the program itself [18]. Also, these studies related to traditionally delivered exercise regimes and did not specifically focus on newer technology-based alternatives.

A survey-based study evaluated technology-based health behavior interventions versus traditional modalities [22] in cancer survivors. This indicated a receptivity to using Web apps as a technological delivery medium. An online questionnaire-based study in cancer survivors evaluated preferences for technology-supported exercise interventions and indicated they may be feasible and acceptable [23]. It would appear that no prior study has integrated in-depth personalized insights of eHealth-based PA interventions at the pretrial phase to inform the design of such an intervention in cancer survivors. The overall aim of this study was to explore perspectives of cancer survivors toward the concept of an eHealth-based PA program. To address this aim, a phased approach was taken.

This paper will briefly describe a preliminary questionnaire-based study to ascertain basic information pertaining to self-reported PA levels, knowledge of PA guidelines, smartphone use, as well as interest in a follow-on focus group study. The main focus of the paper will be a focus group study that qualitatively explores perspectives of cancer survivors toward the concept of an eHealth-based PA program.

## Methods

### Overview

The preceding questionnaire-based study will be described first, followed by the follow-on focus group study. Both studies took place in St. James’s Hospital, Dublin, Ireland, an acute-care hospital that is one of the largest designated cancer centers in Ireland. Written informed consent was obtained separately for each study. Inclusion criteria were as follows: over 18 years of age, attending oncology outpatient clinics, absence of cognitive disabilities that may hinder following instructions, and patients who had received chemotherapy or radiation therapy for malignancy and had finished a course of treatment or were anticipated to finish their treatment within 3 months. Ethical approval was granted by St. James’s Hospital, Tallaght University Hospital Research Ethics Committee (reference: 2015-05).

### Recruitment

Due to the heterogeneous nature of cancer and its treatment, there were a large number of cancer clinics in St. James’s Hospital Oncology service, including breast, gynecological, colorectal, and lung cancer clinics. The lead investigator liaised with the relevant medical and nursing staff in advance of both studies. The treating physician performed initial eligibility screening and advised whether each patient could be approached for study participation. The lead investigator then approached the patient, provided information about the study, and, if appropriate, obtained written informed consent.

### Preceding Questionnaire-Based Study

Preceding the main focus group study, a cross-sectional study was conducted in mixed cancer outpatient clinics to ascertain possible interest in participating in subsequent eHealth-related studies. Participants filled out this 5-minute paper-based questionnaire (see Multimedia Appendix 1) while waiting for hospital-based cancer-related outpatient appointments. As this was such a new area of focus, a short questionnaire was specifically designed to scope out the following information: (1) knowledge of, and adherence to, PA guidelines as well as quantification of sedentary behavior, (2) smartphone ownership and usage of mobile phone app technology, and (3) willingness to participate in further eHealth-related studies. No prior questionnaire existed that explored the use of technology in PA interventions for cancer survivors; therefore, the questionnaire that was developed was based on an existing PA assessment questionnaire [24] and the specific objectives of this study. Willing participants were subsequently contacted for inclusion in the focus group study.

### Follow-On Focus Group Study

Focus groups were employed in this qualitative study, chosen for their strength in generating new ideas and diverse opinions in a way that would be less accessible in a one-to-one interview [25]. A further advantage of focus group design is that participants can develop ideas through facilitated group-based discussion [26]. The design and reporting of research methods used in this study was informed by the COREQ (COnsolidated criteria for REporting Qualitative research) standardized reporting guidelines [27]. Participants were chosen from the pool of participants in the preceding questionnaire-based study who indicated a willingness to participate in a focus group study. A convenience sampling method was adopted in this study, with participants included being the first who responded and were available for participation.

### Data Collection

Focus groups were conducted by the lead investigator who was also group moderator (CH). CH was a doctoral student with a background as a physiotherapist, who was not involved in the clinical care of participants. He had additional training in qualitative methodology and focus group facilitation. The assistant facilitator varied between two people, depending on availability, and was either an academic (JB) or a postdoctoral researcher (JM), both trained in qualitative methodology. No repeat interviews took place and transcripts were not returned to participants for accuracy.

All interviews were recorded using a Voice Tracer DVT2000 digital recorder (Philips). CH facilitated the discussion and JB or JM took field notes, including observations during the interviews. These field notes assisted in identifying potential themes that emerged that the lead moderator may have missed, as well as recording general observations that assisted in data analysis.

At the start of each focus group, brief study information was provided regarding goals and reasons for conducting this research, and ground rules were agreed upon. An interview guide (see Multimedia Appendix 2) was developed based on prestated study objectives, results of a previous systematic review [12], and relevant qualitative literature [28]. The interview guide was semistructured to encourage a free flow of conversation [29]. The interview guide was not pilot-tested prior to the first focus group. Data collection continued until saturation was reached, a stage where no new ideas or themes emerged [30].

### Data Analysis

Questionnaire data from the first study was analyzed descriptively. In the focus group study, to optimize rigor, a synopsis of the main points was given at the conclusion of each focus group, whereby participants were questioned regarding whether it was an accurate portrayal of what had been discussed.

In view of the emergent nature of this area, data analysis was performed using thematic analysis following the phased approach outlined by Braun and Clarke [31]. Recordings were transcribed verbatim by CH and double-checked for accuracy by JB. Focus group transcripts were coded into meaningful clusters using NVivo 9 (QSR International) qualitative data analysis software. Two independent researchers (CH and JM) performed this inductive coding and produced a collection of codes that they deemed to have meaning in the context of the stated objectives of the focus groups. The data were examined to establish recurring patterns of meaning. Codes and themes were discussed, refined, and agreed upon by authors and then checked and compared to ensure grouped data were contextually meaningful. Any differences in coding were discussed by researchers until a consensus was achieved.

## Results

### Results of Preceding Questionnaire-Based Study

This study took place between August 2015 and January 2016 and included 102 participants. Due to the nature of our method of recruitment, there were no refusals to participate once the patients were referred to the lead investigator from their treating physicians. There were slightly more female participants included in the study (54/102, 52.9%). The mean age of the participants was 65.5 years (SD 14.3).

Participants had a range of cancer diagnoses, with the highest number having colorectal cancer (52/102, 51.0%). Results indicated that almost half (46/102, 45.1%) of all participants reported to be achieving or exceeding guideline PA levels. A total of 63.7% (65/102) of participants overestimated the recommended weekly PA, while 18.6% (19/102) underestimated the guideline for weekly PA.

The number of smartphone users was 59.8% (61/102), with lower numbers noted in those over 65 year of age. It was also identified that 89% (54/61) of those that had access to smartphones used smartphone apps. The most frequently specified mobile apps were Facebook (14/61, 23%) and WhatsApp (9/61, 15%). Only 16% (10/61) of participants reported using PA or exercise apps on their smartphones.

Interest in participating in a follow-on focus group was expressed by 61% (37/61) of participants who owned or had access to a smartphone. Interest in participating in a future eHealth PA intervention was also high (47/61, 77%) among participants in this study.

### Results of the Focus Group Study

Seven focus groups were conducted between November 2015 and April 2016. In total, six focus groups had 3 participants present, with one focus group having 5 participants present. Data saturation was reached following analysis of the sixth and seventh focus groups. This resulted in conclusion of the study after the seventh focus group, with a final sample size of 23 participants. The remaining 14 participants who expressed interest in participating in the focus groups could not attend after they were recontacted; reasons given were mostly due to weather, being unwell on the day of the focus group, lack of interest, and having difficulty accessing the center due to travel distance.

### Focus Group Participant Characteristics

Demographic details of the participants are collated in Table 1. The focus groups ranged from 23 to 34 minutes in length and the mean duration of the focus groups was 28.7 minutes (SD 3.4). Out of 23 participants, 17 were female (74%) and 6 were male (26%), and they had a mix of cancer diagnoses. The age range was 34-82 years. Out of 23 participants, 12 were over 65 years of age (52%) and 11 were 64 years of age or under (48%).

**Table 1:** Demographic details of focus group participants.

Variable		Value (n=23)
**Gender, n (%)**		
	Male	6 (26)
	Female	17 (74)
Age at study enrollment (years), mean (SD)		61.34 (12.60)
**Cancer type, n (%)**		
	Breast	4 (17)
	Colorectal	6 (26)
	Ovarian	5 (22)
	Testicular	2 (9)
	Endometrial	3 (13)
	Other	3 (13)
**Treatment, n (%)**		
	Chemotherapy only	15 (65)
	Chemotherapy and radiotherapy	8 (35)
	Surgery	23 (100)
**Marital status, n (%)**		
	Married	14 (61)
	Single	9 (39)

### Results of Thematic Analysis of Focus Groups

#### Overview

**Figure 1:**
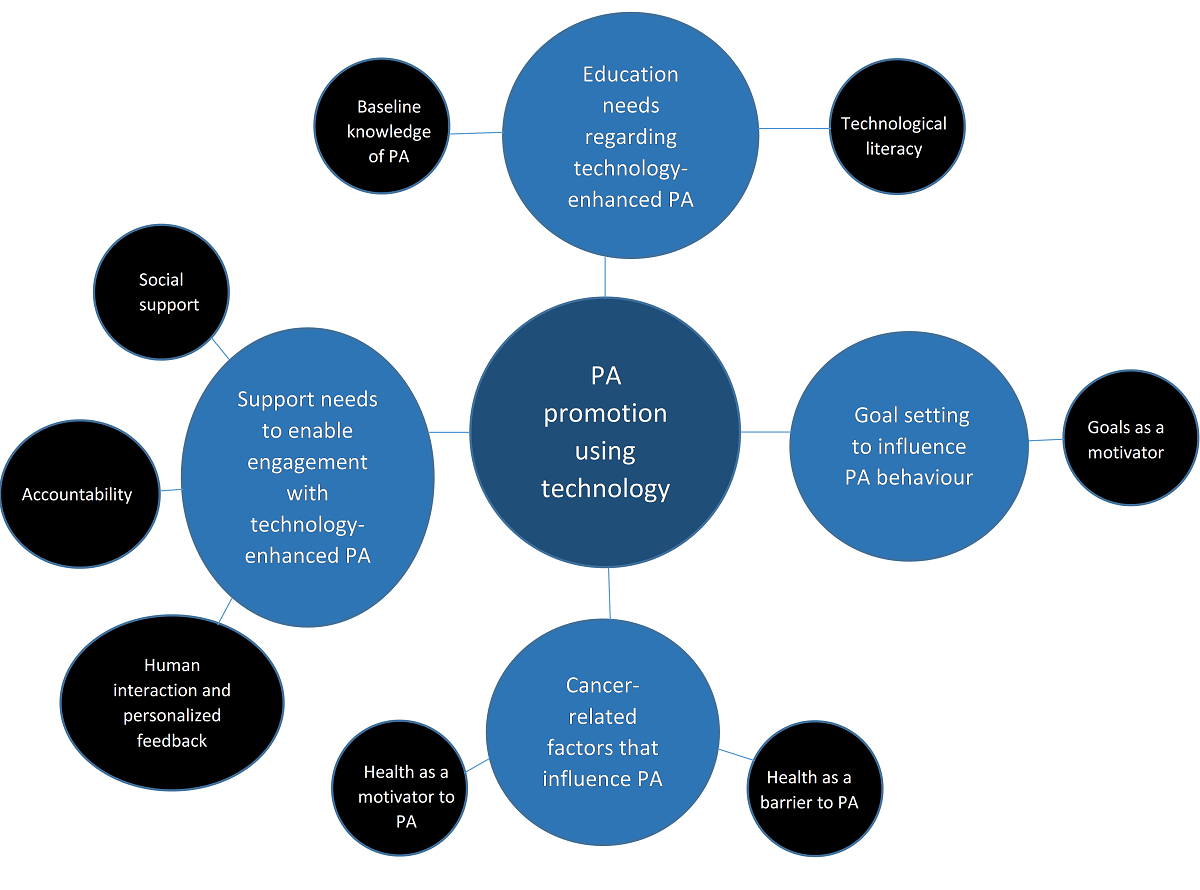
Themes and subthemes following analysis and coding of focus group transcripts. PA: physical activity.

Following analysis and coding of the transcripts, a number of themes and subthemes were generated from the data and are detailed in Figure 1. There were four main themes—*health impact*, *education needs*, *support needs*, and *goal setting*—with accompanying subthemes. The role of technology was embedded throughout these themes.

#### Theme 1: Health Impact

##### Overview

The initial opening question “What motivates you to exercise?” generated discussion around general PA-related factors. A strong generic theme that was generated was the topic of *health impact*. Two distinct subthemes generated from this main theme were the role of health as a barrier to PA and, conversely, its role as a facilitating factor or motivator for PA.

##### Subtheme 1: Health as a Barrier to Physical Activity

There were a number of participants who signaled that side effects of cancer treatment or general health were primary barriers to PA, with fatigue frequently referenced.

Ever since the chemo I’ve lost interest ... got so tired.

Participant #80, female, 76 years, ovarian cancer

##### Subtheme 2: Health as a Motivator to Increase Physical Activity

Some participants remarked that good health and feeling better were motivating factors to increase PA.

When I was going through the treatment I felt like going out for a walk, no matter how tired I was ... and I think it helped me through the treatment ... and helped me overall.

Participant #19, female, 65 years, ovarian cancer

Losing weight and improving general fitness served as motivation for a number of participants.

Well mine is to lose weight, and to get a bit fitter.

Participant #85, female, 58 years, breast cancer

I felt that walking before I got sick helped me, kinda get strong you know, helped me you know, physically, helped me through the treatment as well, you know.

Participant #19, female, 65 years, ovarian cancer

#### Theme 2: Education Needs

##### Overview

The theme of education featured prominently in terms of knowledge about PA, technical literacy, and the need for a PA program that features human interaction in tandem with eHealth.

##### Subtheme 3: Baseline Knowledge of Physical Activity

Throughout the focus groups, the absence of education about the importance of PA following a cancer diagnosis was frequently discussed.

After the treatment I was never really told exercise was important.

Participant #56, female, 53 years, colon cancer

I didn’t hear anything about it at all, until I met you [speaking to lead investigator].

Participant #57, female, 60 years, breast cancer

Even if you got a leaflet, even if there was something, or the book recommend, a book to read, but there was nothing.

Participant #69, male, 76 years, rectal cancer

##### Subtheme 4: Technical Literacy

Education about technology and technological literacy also presented under the umbrella of education. Many participants indicated that for technology to be introduced, they would require education or training on how to use it first.

Someone just to sit there with you, for just a certain amount of time, till you sort of grasp it.

Participant #87, female, 74 years, ovarian cancer

There was also an awareness among some participants that they were not entirely comfortable using technology currently, but they similarly agreed that support and education would make it possible to try using technology.

There’s no point saying to somebody that’s ... never used an app before, “switch on that app and away you go,” you know it’s not as easy as that.

Participant #12, female, 54 years, ovarian cancer

##### Subtheme 5: Built-In Personalization and Feedback

The focus groups highlighted the importance of direction and feedback throughout the program.

I think I’d want a bit of feedback from the like of you [speaking to CH], somebody like you, you know even to keep contact maybe every two weeks.

Participant #38, female, 69 years, rectal cancer

Personalization and the provision of PA prescription specific to each individual participant also became evident.

I think each person is an individual, so no one app, do you know, it has to be adaptable to every single person not just one type of person, so like [Participant #11] said, you input your information there and ... it’s specifically for you, so I think that’s important.

Participant #01, male, 34 years, testicular cancer

#### Theme 3: Goal Setting

##### Overview

One of the main themes to be generated from the data extracted from the focus groups was the concept of goals.

##### Subtheme 6: Goals as Motivation

The importance of goals was expressed in a number of different ways; however, the role of goal setting as a factor for motivation was particularly prevalent. Implicit in this theme was the concept of self-monitoring.

I’ll say, “hey that’s not good enough now ... I’m definitely going to go 2 km and then I’ll get to 2,” and I’ll think, “ah sure I’m at 2 now, I don’t feel so bad, maybe I’ll go to 3,” and then it actually motivated me every day to beat my previous record.

Participant #12, female, 54 years, ovarian cancer

When participants were asked whether having a smartphone app could help improve PA, one participant who had been using an app agreed that it did, again highlighting the presence of a target or goal as a motivator.

Yeah, it did, because I had a target, tell you exactly what you’ve done, if you’ve hit that target, well not every day, but maybe once a week, trying to beat that target.

Participant #81, male, 57 years, esophageal cancer

#### Theme 4: Support Needs

##### Overview

The theme of support featured prominently and was heavily discussed. It took the form of two subthemes that resulted from the analysis: accountability and social support.

##### Subtheme 7: Accountability

The importance of accountability, so participants would be *answerable* to an individual, was evident.

Well even just to sit, and talk to somebody like yourself, and to feel like there is somebody there, that you care if we do exercise or not.

Participant #87, female, 74 years, ovarian cancer

##### Subtheme 8: Social Support

In contrast to the professional, prescriptive support that participants mentioned as important, the majority of participants also described motivation stemming from family, friends, and peers.

My friends and family more so, kind of influence in a way, they say, “I’m going for a walk, do you want to go for a walk?” I’ll say, “yeah, sure why not.”

Participant #01, male, 34, testicular cancer

### Application of Findings to Intervention Design

A summary of the technological features from the themes and subthemes to be integrated into the eHealth PA-based intervention are listed in Textbox 1.

**Key design features of eHealth-based physical activity interventions.**

- Personalized instruction to upskill technical literacy
- Integrated education about physical activity
- Integrated goal setting
- Integrate peer support where possible
- Tailored program—individually prescribed
- Blended program, including technology and human interaction and personalized professional guidance throughout the program
- Supervision for initial session
- Feedback on behavior
- User friendly

## Discussion

### Principal Findings

eHealth-based PA interventions are an emerging type of intervention for cancer survivors. The aim of this study was to explore perspectives of cancer survivors toward the concept of an eHealth-based PA program. The initial scoping study highlighted the lack of knowledge of PA guidelines, which echoes the focus group findings. PA levels were likely to be overestimated due to the crude self-report method of quantification [32]. The majority of participants were familiar with and used mobile apps, but usage of health-focused apps was low. This questionnaire-based study provided useful preparatory research for the design of the subsequent focus group study, and a high level of interest to participate in future eHealth-based studies was shown in this sample.

This focus group study delved much deeper into this topic and showed that while receptivity to the concept of an eHealth-based intervention was positive, participants need integrated education about the role of PA, technological upskilling to enable engagement with this medium, and some face-to-face interaction with a health professional in tandem with the remotely delivered aspect of an eHealth program.

This study highlighted the need for face-to-face support to initialize patients at the start of an eHealth program. The value of a trusted patient–health care provider relationship has been highlighted in a study that evaluated perspectives of mHealth interventions (ie, health interventions supported by a mobile device) in cancer survivors [33] and in patients with rheumatoid arthritis [34]. Our study showed that instead of a fully automated eHealth program, a blended program with personalized and formalized face-to-face human interaction integrated with eHealth would be optimal, which echoes previously identified program preferences [34].

Perspectives from this study indicated that an important technical specification to incorporate is personalized goal setting. Goal setting has previously been identified in a focus group study of cancer survivors as important in helping promote increased PA levels [35] and is underpinned by a well-recognized theoretical framework [36]. Further behavior change techniques that should be incorporated are feedback on behavior—automated and personalized—as well as self-monitoring, mirroring work from a recent study [33].

Peer support as an important element of group-based interventions was also referenced in this study, which mirrors previous research [37-39]. It has been suggested that the group dynamic enables better emotional support and coping skills than mediums that are not face-to-face, such as websites or books [38]. Conversely, a large qualitative study of cancer survivors’ perspectives of a cancer rehabilitation program indicated that participants were not motivated by the group aspect per se and risked dependency [39], so transitioning to “real life” outside the intervention can be difficult. Notably, practical challenges of integrating group-based exercise outside the home setting, such as travel and scheduling challenges [39], are overcome by eHealth-based interventions. Nonetheless, as peer support came across as an important motivational element from the perspective of cancer survivors in this study, we suggest integrating this into eHealth programs where possible (see Textbox 1).

Several participants identified a technological training need to upskill sufficiently to enable engagement with eHealth-based interventions due to low confidence in their computer literacy. This lack of knowledge of technology was not the only deficit highlighted by this study, with results from the preceding questionnaire study highlighting a lack of knowledge of optimum PA, with only 17.6% (18/102) of participants correctly identifying recommended PA guidelines as identified by the American Cancer Society [40]. Creating an opportunity for health professionals to bring up the benefits of PA and methods to improve PA behaviors is needed. Exercise preferences were not explored in this study, but it was implicitly stated throughout that walking was the most preferable form of exercise, which mirrors similar research in cancer survivors [33]. Building strength and flexibility in cancer survivors is also valuable [40], and it would be important to incorporate other modes of exercise in an eHealth-based PA program.

A number of strengths pertained to these two studies. The initial questionnaire-based study indicated a receptivity to further eHealth-based studies, which is likely important to establish in a new area of focus such as this. In the focus group study, participants were not biased by a predetermined program. This study involved identifying end users’ needs and preferences to inspire and influence the technological aspects of the intervention, which can be applied to the design of future interventions. This study provided valuable information on acceptability and intervention components [15].

Focus groups conducted at the pretrial phase have an added value that can optimize the design of the intervention and trial procedures [15]. Employing focus groups provided the opportunity to drill down and generate a depth of information not found in the preceding cross-sectional questionnaire-based study. There was a small number of participants in each focus group, which we observed to be less intimidating [6] and encouraged interaction, although it may potentially have limited diversity of views.

### Study Limitations

Resource constraints meant the research could be conducted in only one center, although a geographical spread of participants was noted. The generalizability of results to other settings is not known, although we have no evidence to suggest perspectives of this cohort are at odds with other locations. It should also be noted that sample size for the questionnaire study was small and may not be representative of the cancer survivor population. The mean age was over 60 years and participants were predominately female (74%) which may have influenced the results of the focus group. Naturally, in a heterogeneous disease such as cancer, it is likely that design of an eHealth intervention should be nuanced with a need for different considerations, such as increased supervision for people with advanced and metastatic diseases [33] and for those with a range of comorbidities. Ideally, a suite of PA options should be available to cancer survivors, of which eHealth appears to be an acceptable option. Also, an inherent limitation of this pretrial focus group study is that a hypothetical PA program was discussed, which may have given rise to overly positive comments due to social desirability bias [41].

### Clinical Implications

An important consideration in the design of eHealth-based interventions for people with cancer is to consider that technological upskilling may be necessary to bridge the knowledge gap and ease initial trepidations to optimally harness the potential of this medium. Opportunities for interaction with a health care provider need to be built into the program. The program should be individualized, and essential behavior change elements to integrate into the program are goal setting and feedback on behavior.

### Future Directions

Future qualitative work should include other stakeholder perspectives and evaluation of user experience after completion of the eHealth interventions. There was a notable absence of issues relating to privacy and data security in the focus groups. Other behavioral change techniques, such as prompts and cues to be more physically active as well as incentives, rewards, and gamification, were not raised by participants but response to these behavioral change techniques may be mixed [33]. Future studies should nonetheless explore these pertinent topics.

Stakeholder perspectives gleaned from this study have informed key design features of the IMPETUS (IMproving Physical activity and Exercise with Technology Use in cancer Survivors) trial (ClinicalTrials.gov registration: NCT03036436), which we have recently conducted in our center. The intervention was based on intervention elements summarized in Textbox 1 and underpinned by sound behavioral change theory [36], which included aspects of goal setting, prompts, self-monitoring, and encouragement of independent exercise. Findings reported in this paper will help design and reconfigure future interventions incorporating this new and exciting medium.

### Conclusions

Given recent advancements that offer more technologically enhanced programs, this type of research is warranted to tailor design features and optimize their acceptability to cancer survivors. Even though low levels of technological literacy were reported among some participants, it would appear that there is an initial receptivity to the concept of eHealth-based PA interventions. However, these interventions should not be delivered in isolation, but with technological upskilling, built-in human interaction, and integrated behavioral change techniques in tandem. This study will add to the body of literature to ensure that eHealth interventions are user informed and tailored to suit the unique needs of cancer survivors.

## Appendix

Multimedia Appendix 1 Questionnaire.

Multimedia Appendix 2 Interview guide for focus groups.

## Abbreviations

COREQ: COnsolidated criteria for REporting Qualitative research
eHealth: electronic health
IMPETUS: IMproving Physical activity and Exercise with Technology Use in cancer Survivors
mHealth: mobile health
MRC: Medical Research Council
PA: physical activity
