# Supplementary material for: Perceptions of eHealth-Enabled Physical Activity Interventions Among Cancer Survivors: Mixed Methods Study
Source: JMIR Cancer. 2020 Apr 28;6(1):e16469. doi: 10.2196/16469 (PMC7218594; doi:10.2196/16469)
Supplement: Multimedia Appendix 1 [file cancer_v6i1e16469_app1.pdf]

Trinity College Dublin
Coláiste na Tríonóide, Baile Átha Cliath
The University of Dublin

Questionnaire

Title: *Exploration of the possible use of mobile technology to promote physical activity in patients with cancer.*

This questionnaire will only take 5 to 10 minutes to complete. Most of the questions ask you to answer YES or NO next to your question. All questionnaires will be treated with the strictest confidence.

There is a research physiotherapist available if you have any questions or would prefer someone to fill the form in with you.

More information on this questionnaire is available in the information leaflet you have been given. You may also contact Ciaran Haberlin if you have further queries. Email: haberlic@tcd.ie Phone: 0852432679

Questionnaire

1. How many days a week of physical activity or exercise are recommended for the average adult to stay healthy?

   ____________________ (number of days)

2. On those days, how long should the average adult be physically active to stay healthy?

   ___________________ (minutes or hours)

3. In the past week, on how many days have you done a total of 30 minutes or more of physical activity, which was enough to raise your breathing rate. This may include sport, exercise, and brisk walking or cycling for recreation or to get to and from places, but should not include housework or physical activity that may be part of your job

0 days □
1 day □
2 days □
3 days □
4 days □
5 days □
6 days □
7 days □

4. On average how many hours per day do you spend sitting or lying during waking hours?

   ___________________ (hours)

5. Do you own or have access to a smartphone?

6. Do you use smartphone applications?

7. What is your most commonly used application?

8. Do you use any physical activity or exercise applications on your smartphone?

9. Would you be interested in taking part in a group discussion about improving your physical activity using a smartphone application?

10. Would you be interested in taking part in a research study which will investigate the effect of smartphone applications on your daily physical activity?
