# Supplementary material for: Perceptions of eHealth-Enabled Physical Activity Interventions Among Cancer Survivors: Mixed Methods Study
Source: JMIR Cancer. 2020 Apr 28;6(1):e16469. doi: 10.2196/16469 (PMC7218594; doi:10.2196/16469)
Supplement: Multimedia Appendix 2 [file cancer_v6i1e16469_app2.docx]

*Interview Guide*

**Introduction:**

Participants will be welcomed and the purpose of the focus group will be explained again. Light refreshments will be served at this point. This will act as an ice-breaker. The moderator and the co-moderator will be introduced.

‘I’d like to welcome you all to this focus group discussion. Thanks for agreeing to be part of the focus group. My name is Ciaran Haberlin and I will be leading the discussion, my supervisor Julie Broderick will be taking notes throughout. We appreciate your willingness to participate in this discussion today’.

Our topic is the promotion of physical activity using mobile technology/smartphones. What we are trying to find out, is if there is a way to encourage people to exercise more using smartphones or other technologies.

**Ground rules**

**No right or wrong answers**

**We are tape recording- one person to speak at a time**

**First name basis**

**You don’t need to agree, but listen respectfully to each other**

**Phones to be turned off, or if you must take a call please do so quietly, away from the group.**

**My job will be to guide the discussion**

**Start recording**

This is focus group number 1

**Subject Questions:**

**What motivates you to exercise?**

*Probing question: Is there anything in particular that could help?*

**Do you think a smartphone application could help you to increase your daily physical activity?**

*Probing Question: In what way could it do this? What features would be useful? Why would it not help?*

**Would anything stop you from using mobile technology to help you to exercise more?**

*Probing question: Can you explain these barriers?*

**Can you think of any ways you could overcome these difficulties?**

*Probing questions: How would these solutions work to make mobile technology effective in helping you exercise?*

**Is there anything that would make it easier to use mobile technology?**

*Probing questions: What support/help do you think would facilitate you to use a smartphone application in physical promotion?*

**Are there any smartphone app features that you think would help you to exercise more?**

**Clarifying Questions:**

Can you expand a little on this? Can you give some examples?

**Note taker question/observation**

Any theme that was not elaborated sufficiently, any question that could be asked.

**Closing Questions**

**Asked of each participant: Of everything we talked about today, what to you is the most important part?**

Is there any other information regarding your experience with mobile technology and exercise that you think would be useful for me to know?
